# *Armillifer armillatus* Pentastomiasis in African Immigrant, Germany

**DOI:** 10.3201/eid1903.121508

**Published:** 2013-03

**Authors:** Dennis Tappe, Alexandra Haeupler, Hansjörg Schäfer, Paul Racz, Jakob P. Cramer, Sven Poppert

**Affiliations:** Author affiliations: University of Würzburg, Würzburg, Germany (D. Tappe);; Bernhard-Nocht-Institute for Tropical Medicine, Hamburg, Germany (A. Haeupler, P. Racz, S. Poppert);; University Medical Center Hamburg–Eppendorf, Hamburg (H. Schäfer, J.P. Cramer)

**Keywords:** Armillifer armillatus, pentastome, parasites, pentastomiasis, PCR, formalin fixation, immigrant, zoonoses, Germany, Togo

**To the Editor:** Pentastomiasis is a parasitic zoonotic disease with an incremental number of reported human infections caused by larval stages (nymphs) of pentastomes (*1**–**3*). The vermiform parasites are in their own phylum and are related to branchiuran crustaceans (*2*). Most human infections with these parasites are caused by *Armillifer armillatus* (*2*), a parasite endemic to western and central Africa. Most cases are reported from the Congo region and Nigeria, and occasionally infections in African immigrants to Europe and North America have been reported (*4**,**5*). Imported cases to Germany have not been reported. *A. grandis*, a related parasite from central Africa, has been rarely found (*6*), but *A. moniliformis*, a pentastome species from Asia, has recently reemerged and caused a human infection after ≈40 years in Malaysia (*1*).

Adult *Armillifer* spp. inhabit the respiratory tract of large snakes (*Python* spp.). These dioecious parasites produce large amounts of ova that are shed into the environment by snake feces and secretions. When intermediate hosts, such as rodents or other small mammals, ingest ova, larvae hatch, migrate to the viscera, encyst, and molt several times (*3*). Humans become accidental intermediate hosts after uptake of environmental parasite ova or by consumption of contaminated snake meat. We report an infection with *A*. *armillatus* in an African immigrant to Germany that was diagnosed by histopathologic analysis and confirmed by PCR.

In 2005, a 23-year-old man from Togo who had immigrated to Germany 3 years earlier showed development of acute myeloid leukemia. He subsequently underwent stem cell transplantation, which was followed by graft versus host disease. The patient died of sudden intracerebral hemorrhage and leukencephalopathy. His medical history also included α-thalassemia and a heterocygotous sickle cell trait, chronic hemolytic anemia, splenomegaly, and cardiomyopathy. He had been treated for schistosomiasis and filariasis.

An autopsy specimen showed several living pentastome nymphs of ≈2 cm in size, which were found in the subscapular region of liver parenchyma. A presumptive diagnosis of visceral pentastomiasis caused by *A. armillatus* nymphs was made in accordance with the origin of the patient and the geographic distribution of the parasite.

Microscopic slides from patient specimens were retrieved from an archive and reanalyzed (Figure, Technical Appendix Figure). A pentastome-specific PCR targeting the 18S rRNA gene (*2**,**7*) was conducted after DNA extraction from formalin-fixed tissue on a remaining unstained microscope slide. The resulting 383-bp amplicon was sequenced, and BLAST analysis (www.ncbi.nlm.hih.gov/blast) confirmed 100% identity with *A*. *armillatus* (GenBank accession no. HM756289.1) and 99% homology with *A*. *agkistrodontis* (FJ607339.1) and *A*. *moniliformis* (HM048870.1).

**Figure:**
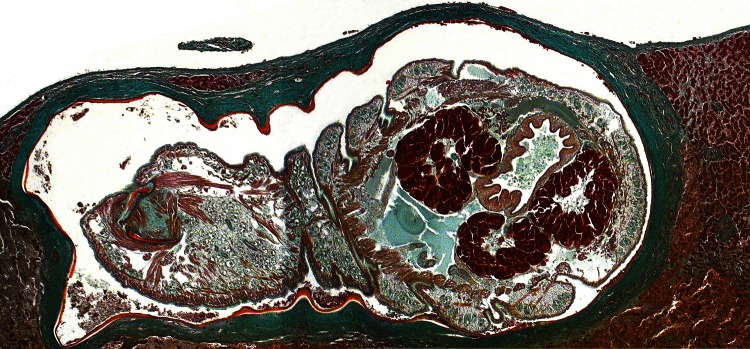
Oblique cross-section of liver of a patient (immigrant) from Togo, showing a well-preserved *Armillifer armillatus* nymph in a subcapsular location. The annulated parasite is encapsulated by its shed cuticle (exuvia) and dense fibrosis. Consistent with the viable type of a pentastomid lesion (*3*), no inflammatory infiltrate is visible. This image also shows internal structures of the pentastome, such as prominent bunches of acidophilic glands surrounding the intestine (Masson’s trichrome stain, original magnification ×10).

Visceral pentastomiasis in humans is often asymptomatic and an incidental finding during surgery (*1**,**3*) or autopsy (*8**,**9*). In a large autopsy series from Malaysia, a pentastomiasis prevalence of 45.5% was found in adult Aborigines (*8*). In Nigeria, a rate of 33% was seen during autopsies of patients who had died of malignancies (*9*). However, a few severe and even lethal cases have been described for heavy *A. armillatus* and *A*. *grandis* infections in persons from Africa (*4**,**6*).

Diagnosis is achieved by gross pathologic and histopathologic analyses. Nymphs are found in the serosa around the liver and spleen, in liver parenchyma, mesenterium, intestine wall, and abdominal lymph nodes. The lungs or pleura are occasionally infected (*3*). Radiographic analysis may show typical C-shaped chest or abdominal calcifications (*10*). Species identification is performed by counting annulations (*A*. *armillatus* 18–22, *A*. *grandis* >25) and measuring the size of larval parasites (*3*). Recently, PCR has been used for diagnosis in veterinary infections (*2**,**7*).

For the patient in our study, molecular analysis identified human pentastomiasis by using a formalin-fixed microscope slide that had been stored for 7 years. A difference of 2 nt each was seen when the amplified nucleotide sequence was compared with database sequences of *A*. *agkistrodontis* and *A*. *moniliformis*. However, there is no database entry in GenBank for *A*. *grandis*, the geographically closest *Armillifer* species. Serologic assays have been developed for identification of *A*. *armillatus* (*2*), but no serum was available for retrospective analysis. In special settings, such as tropical snake farming and pet keeping, pentastomiasis may be a public health concern (*2*). However, most infections have been linked to consumption of undercooked snake meat or other snake products (*1*). 

Most immigrants who were given a diagnosis of visceral pentastomiasis were from Nigeria or the Congo region, and diagnoses were made after death. Molecular analysis is particularly valuable when only autoptic paraffin-embedded patient material is available. For industrialized countries, where experience in morphologic identification of unusual parasite species is limited, molecular analysis is a valuable diagnostic tool. Our case-patient constitutes a record of imported *Armillifer* species pentastomiasis to Germany. Because of increasing international migration, more cases of pentastomiasis are likely to be seen.

Technical Appendix Figure Transverse section of an *Armillifer armillatus* larva from the liver of a patient (immigrant) from Togo. Typical for pentastomid lesions, the parenchyma shows focal hemorrhage around the parasite and no inflammatory cellular reaction (*1*). There is also focal destruction of the trabecular liver parenchyma. Subcuticular gland cells of the parasite are visible, and the intestine is clearly discernable in the center (hematoxylin and eosin stain, original magnification ×20).
