# Supplementary material for: Armillifer armillatus Pentastomiasis in African Immigrant, Germany
Source: Emerg Infect Dis. 2013 Mar;19(3):507–8. doi: 10.3201/eid1903.121508 (PMC3647675; doi:10.3201/eid1903.121508)
Supplement: Technical Appendix Figure — Transverse section of an Armillifer armillatus larva from the liver of a patient (immigrant) from Togo. Typical for pentastomid lesions, the parenchyma shows focal hemorrhage around the parasite and no inflammatory cellular reaction (1). There is also focal destruction of the trabecular liver parenchyma. Subcuticular gland cells of the parasite are visible, and the intestine is clearly discernable in the center (hematoxylin and eosin stain, original magnification ×20). [file 12-1508-Techapp-s1.pdf]

Article DOI: http://dx.doi.org/10.3201/eid1903.121508

Armillifer armillatus Pentastomiasis in African Immigrant, Germany

Technical Appendix

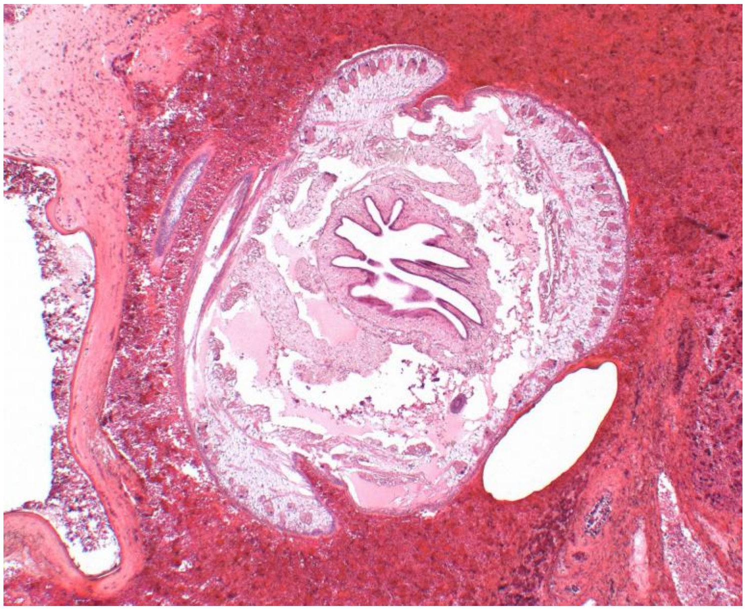

Figure. Transverse section of an *Armillifer armillatus* larva from the liver of the patient (immigrant) from Togo. Typical for pentastomid lesions, the parenchyma shows focal hemorrhage around the parasite and no inflammatory cellular reaction (*1*). There is also focal destruction of the trabecular liver parenchyma. Subcuticular gland cells of the parasite are visible, and the intestine is clearly discernable in the center (hematoxylin and eosin stain, original magnification ×20).

Reference

1. Tappe D, Büttner DW. Diagnosis of human visceral pentastomiasis. PLoS Negl Trop Dis. 2009;3:e320. PubMed http://dx.doi.org/10.1371/journal.pntd.0000320
